# Automating Rey Complex Figure Test scoring using a deep learning-based approach: a potential large-scale screening tool for cognitive decline

**DOI:** 10.1186/s13195-023-01283-w

**Published:** 2023-08-30

**Authors:** Jun Young Park, Eun Hyun Seo, Hyung-Jun Yoon, Sungho Won, Kun Ho Lee

**Affiliations:** 1 Gwangju Alzheimer’s & Related Dementia Cohort Research Center, Chosun University, Gwangju, 61452 South Korea; 2 Department of Public Health Sciences, Graduate School of Public Health, Seoul National University, Seoul, 08826 South Korea; 3 Neurozen Inc., Seoul, 06168 South Korea; 4 Premedical Science, College of Medicine, Chosun University, Gwangju, South Korea; 5 Department of Neuropsychiatry, College of Medicine, Chosun University, Gwangju, South Korea; 6 Interdisciplinary Program in Bioinformatics, Seoul National University, Seoul, South Korea; 7 Institute of Health and Environment, Seoul National University, Seoul, South Korea; 8 RexSoft Inc., Seoul, 08826 South Korea; 9 Department of Biomedical Science, Chosun University, Gwangju, South Korea; 10 Korea Brain Research Institute, Daegu, 41062 South Korea

**Keywords:** Alzheimer’s disease, Rey Complex Figure Test, Scoring, Artificial intelligence, Deep learning, Convolutional neural network

## Abstract

**Background:**

The Rey Complex Figure Test (RCFT) has been widely used to evaluate the neurocognitive functions in various clinical groups with a broad range of ages. However, despite its usefulness, the scoring method is as complex as the figure. Such a complicated scoring system can lead to the risk of reducing the extent of agreement among raters. Although several attempts have been made to use RCFT in clinical settings in a digitalized format, little attention has been given to develop direct automatic scoring that is comparable to experienced psychologists. Therefore, we aimed to develop an artificial intelligence (AI) scoring system for RCFT using a deep learning (DL) algorithm and confirmed its validity.

**Methods:**

A total of 6680 subjects were enrolled in the Gwangju Alzheimer’s and Related Dementia cohort registry, Korea, from January 2015 to June 2021. We obtained 20,040 scanned images using three images per subject (copy, immediate recall, and delayed recall) and scores rated by 32 experienced psychologists. We trained the automated scoring system using the DenseNet architecture. To increase the model performance, we improved the quality of training data by re-examining some images with poor results (mean absolute error (MAE) $\ge$ 5 [points]) and re-trained our model. Finally, we conducted an external validation with 150 images scored by five experienced psychologists.

**Results:**

For fivefold cross-validation, our first model obtained MAE = 1.24 [points] and *R*-squared (${R}^{2}$) = 0.977. However, after evaluating and updating the model, the performance of the final model was improved (MAE = 0.95 [points], ${R}^{2}$ = 0.986). Predicted scores among cognitively normal, mild cognitive impairment, and dementia were significantly different. For the 150 independent test sets, the MAE and ${R}^{2}$ between AI and average scores by five human experts were 0.64 [points] and 0.994, respectively.

**Conclusion:**

We concluded that there was no fundamental difference between the rating scores of experienced psychologists and those of our AI scoring system. We expect that our AI psychologist will be able to contribute to screen the early stages of Alzheimer’s disease pathology in medical checkup centers or large-scale community-based research institutes in a faster and cost-effective way.

## Background

The Rey Complex Figure Test (RCFT) was originally developed to evaluate the perceptual organization and visual memory [1]. It is valuable and practical in that the test is relatively simple and clear to administer, and it assesses multiple cognitive domains, including executive function and visuospatial ability or memory [1, 2]. The RCFT has been widely used to evaluate the neurocognitive functions in various clinical groups with a broad range of ages [3, 4]. Visuospatial modality of episodic memory has been suggested as having a significant association with tau pathology in Alzheimer’s disease (AD) [5–8]. Particularly, previous studies on Alzheimer’s continuum have demonstrated that RCFT scores can be an early marker of clinical progression [9] or tau pathology [5]. In addition, the RCFT sensitively captures organizational strategies in healthy young adults [10] and in patients with brain damage [11, 12].

Several quantitative and qualitative scoring systems have been proposed [3]. The most broadly used method is the 18-item and 36-point scoring system standardized by Osterrieth [13]. However, despite its usefulness, the scoring method is as complex as the figure. Complex scoring system could lead to the risk of reducing the extent of agreement among raters [14]. Therefore, it is essential to acquire scoring skills before the administration of the RCFT. Raters for the RCFT need to be trained intensively to score reliably. Consequently, conducting the RCFT in large-scale community-based studies is difficult. However, the demand for digital-based cognitive assessments has increased. The digitalization of cognitive assessment has developed rapidly with technological advancements [15]. Traditional cognitive measures such as the RCFT are reliable candidates for digitalization. Establishing an automatic scoring system for the RCFT could be an unavoidable initial step in the evolution of digital cognitive assessments.

Recently, as deep learning (DL) has undergone remarkable improvements in health care [16], there have been some efforts toward automating the assessment of digitalized drawing tests such as the pentagon drawing test (PDT) and the clock drawing test (CDT). In particular, convolutional neural networks (CNN) that can extract important features automatically from raw data have been widely used and shown excellent performance. Several automatic scoring systems for PDT were developed using CNN [17–19]. Previous studies on digitalized CDT have shown that CNN could distinguish subjects with cognitive impairment from cognitively normal (CN) subjects [4, 20, 21].

Meanwhile, for digitalized RCFT, many studies using computer vision technology have been proposed. For instance, digital tablets and pens have been widely used to generate images and extract distinctive features from digitalized images. Hyun et al. [22] showed the differences between adolescents with attention-deficit hyperactivity disorder and healthy adolescents by comparing the pixel mean between a template image and images drawn using a digital tablet. Also, the pen stroke data and spatial information from images drawn by a digital tablet and pen were also used to distinguish subjects with AD from CN subjects [23]. Furthermore, several DL methods were proposed, recently for the digitalized RCFT. CNN methods using raw RCFT images have been applied to differentiate individuals with cognitive impairment from those with CN [24–26]. Those studies have focused on identifying the different patterns between clinical diagnostic groups and healthy controls.

However, methods for directly predicting RCFT scores based on the 36-point scoring system, which is widely used in clinical fields, have been very limited. A method to score the RCFT was firstly developed by segmenting six relevant scoring sections [27]. However, it offered only six of the 18 scoring sections, so could not be applied to the 36-point scoring system. A DL method for scoring the RCFT was proposed [28]. However, they not only did not report detailed information such as sample size and training architecture but also did not have the performance comparable to human experts ($r=0.88$).

If the RCFT scores based on 36 points could be obtained automatically, reliably, and validly, they would be much more flexible to use in various clinical and research settings including AD research. Therefore, we aimed to develop an automated RCFT scoring system based on 36 points using CNN. We selected more than 20,000 drawn RCFT images scored by experienced psychologists and trained the model to predict these scores. To increase the model performance, we improved the quality of the training data by re-examining some images with poor results and developed our final model. Finally, the validity of the predicted scores from our artificial intelligence (AI) system was tested on an independent 150 dataset provided by five experienced psychologists.

## Methods

### Study participants

We included participants enrolled in the Gwangju Alzheimer’s and Related Dementia (GARD) cohort registry at Chosun University in Gwangju, Korea, from January 2015 to June 2021. The overall procedure of the GARD database has been previously described [9]. We selected as many images as possible that had been scored by experienced psychologists.

In total, 6680 subjects, consisting of 4057 CN subjects, 2331 subjects with mild cognitive impairment (MCI), and 292 subjects with dementia, were included in our analyses. We selected all the RCFT scores, including RCFT copy, immediate recall, and delayed recall, and scanned all drawings onto A4 size papers. Finally, we obtained 20,040 scanned images using three images per subject (copy, immediate recall, and delayed recall).

The Institutional Review Boards of Chosun University Hospital and Chonnam National University approved this study. All the participants or their legal guardians provided written informed consent.

### RCFT procedure

The RCFT was administered as one of neuropsychological full batteries to assess visuospatial ability and episodic memory. Full neuropsychological test batteries were introduced in a previous study using the GARD database [5]. The RCFT consists of a copy trial of the complex figure, followed by immediate and delayed recall trials. We selected all three images from the RCFT tests: RCFT copy, immediate recall, and delayed recall. For the RCFT copy, participants were asked to copy the figure on paper without allowing them to rotate either the design or the paper. Erasers were permitted to be used. During the RCFT copy, participants were not given instructions that they would be asked to reproduce the figure from memory. The RCFT immediate recalls were performed immediately after the RCFT copy. RCFT delayed recalls were performed after a 20-min delay. In both cases, participants were asked to draw the figure from memory. There was no time limit to copy or recall the figure. Verbal neuropsychological tests were administered during the delay interval. The scoring method in the present study was applied using the method standardized by Osterrieth [13]. Figures for the RCFT were divided into 18 units, and each unit was scored separately based on the correct place (1 point) and accurate copy (1 point). The sum of the scores for 18 units ranged from 0 to 36. Trained psychologists performed the scoring.

### RCFT image pre-processing

**Fig. 1:**
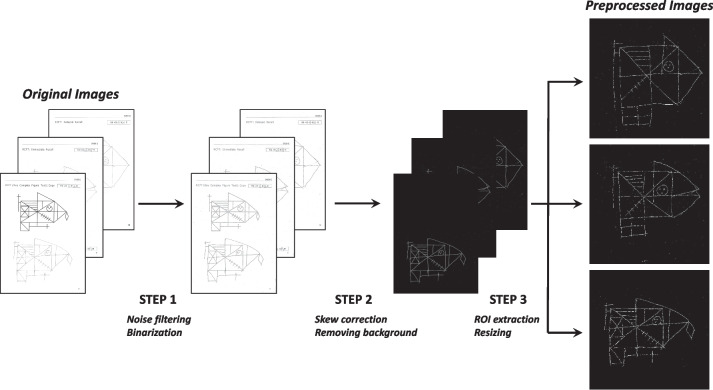
RCFT images pre-processing. First, noise filtering and binarization were applied (step 1). Second, we rotated the images to correct skewness in the process of scanning correction and removed all unrelated background images such as the subject’s name or the number of page (step 2). Third, we obtained the contours of each image to crop the images’ bounding rectangles, and finally, all images were resized to $512\times 512$ pixels (step 3)

RCFT image pre-processing was conducted in three different steps. First, the median filters were applied to smoothen the images. Red–green–blue (RGB) images were converted to grayscale images, and each pixel was dichotomized into either black or white pixels through *adaptive thresholding* (step 1). Second, the scanned images were rotated at different angles and harmonized. Thus, the projection profile method [29] was applied to minimize the effect of image rotation, and all unrelated background images, such as the subject’s name or the number of pages, were removed (step 2). Third, we obtained the contours of each image, and *FindContours* was used to crop the images’ bounding rectangles so that all images were resized to $512\times 512$ pixels, and we finally converted all pre-processed images to RGB scale images to utilize the pre-trained model from the ImageNet Database (step 3). The analyses were conducted using the OpenCV library (version 4.5.5) in Python 3.8. Each pre-processing step is illustrated in Fig. 1.

### DL model

A DL model was developed to score the figures. The proposed model comprised two parts: (1) extracting image features and (2) predicting scores from the extracted features. Spatial information was captured from the images using DenseNet based on the CNN architecture.

**Fig. 2:**
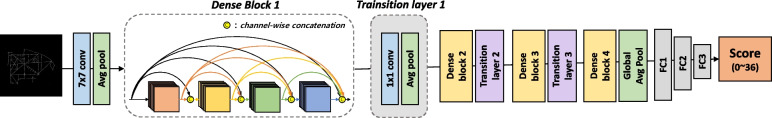
The overall structure of our model. Our model consists of a convolutional block, four dense blocks, three transition layers, a global average pooling layer, and three fully connected layers

The DenseNet [30] architecture was used as the backbone model. Figure 2 shows the overall architecture of the DenseNet model used. It consisted of an initial convolutional block, four dense blocks, three transition layers, a global average pooling (GAP) layer, and three fully connected (FC) layers. Before the first dense block, a convolution block consisted of a convolution layer with a $7\times 7$ kernel size, batch normalization, rectified linear unit (RELU) activation function, and a $2\times 2$ average pooling layer. Each dense block had several convolution networks composed of convolutional layers with $1\times 1$ and 3 $\times 3$ kernel sizes. All convolution networks within each dense block were densely channel-wise concatenated, which improved the information flow. The transition layers were located between dense blocks and consisted of batch normalization, a convolutional layer with a $1\times 1$ kernel size, and a $2\times 2$ average pooling layer, which reduced the dimensions of the features. After the last dense block, the GAP and three FC layers (FC1, FC2, and FC3) were applied. FC1–3 were dense layers of sizes 1000, 128, and 1, respectively, followed by a linear activation function that performed scoring.

Parameter estimation was conducted with the smooth ${\mathcal{L}}_{1}$ loss function because it was less sensitive to outliers than ${\mathcal{L}}_{2}$ loss. We let *n* and *w* be the batch size and parameter vector of the DL model, respectively. The input image and ground truth of the *i*th subject were denoted as ${x}_{i}$ and ${y}_{i}$, respectively. If we let $f\left({x}_{i};w\right)$ be the predicted result from our DL model, the smooth ${\mathcal{L}}_{1}$ loss was defined by:

$${\mathcal{L}}_{\mathrm{smooth}\,L1}= \left\{\begin{array}{c}\frac{1}{n}\sum\limits_{i}{0.5\left(f\left({x}_{i};w\right)-{y}_{i}\right)}^{2}, \ \text{if} \left|f\left({x}_{i};w\right)-{y}_{i}\right|<1, \\ \frac{1}{n}\sum\limits_{i}\left|f\left({x}_{i};w\right)-{y}_{i}\right|-0.5,\ \text{otherwise}\end{array}\right.$$

### Experiments

The performance of the proposed model was evaluated using nested 5-fold cross-validation (CV). In each fold, the training data were split into sub-training and sub-validation data. The ratio of the sub-training and sub-validation data was set to 8:2. Sub-training data were used to optimize the parameters for the proposed model, and the Adam optimizer was adopted to minimize the loss function. We set the base learning rate as 0.001 and decayed the learning rate by multiplying it by 0.1 every five epochs. The initial weights for the CNN were set with pre-trained DenseNet weights in *torchvision.model* from the Pytorch library obtained from the ImageNet database. Early stopping was performed. If the validation loss did not improve in 30 epochs, the training was stopped early to avoid overfitting, and then, the weights with the lowest validation error were selected. Data augmentation was performed by rotating the images at random angles. The rotation degrees were between − 90 and 90° with equal probabilities. Prediction models were built using different folds as training data five times, and the performance was evaluated by concatenating all results of 5-fold. The metrics were mean absolute error (MAE) and *R*-squared (${R}^{2}$). All experiments were conducted using the Pytorch library (v 1.8.1) in Python (v 3.8) using NVIDIA 1080ti GPUs with 48 GB of memory per GPU.

### Evaluating and updating model

The model training consisted of evaluating and updating the model. For the model evaluation step, the prediction model (1st model) with the proposed method was developed, and we evaluated the performance by comparing the ground truths (scores rated by psychologists) and predicted scores (scores that our model rated). The next step was to re-examine some data with errors and update the model using the corrected data. Images with absolute differences larger than five points between the ground truths and predicted scores were checked again and re-examined by experienced psychologists. These were categorized into four different types of errors: image quality error, scoring error, digitalization error, and model bias. Image quality error indicated errors that occurred during the drawing and scanning of images of A4 papers (i.e., eraser trace or damaged papers). Scoring errors indicated inaccurate scoring by psychologists. Errors in the process of digitalizing scores on the computer were called digitalization errors (i.e., typos when digitalizing), and the rest of the errors were considered model biases.

Datasets with errors excepted for model bias were modified to increase the quality of the data. Images with image quality errors were removed from our dataset, and for images with scoring and digitalization errors, we replaced previously ground truths with corrected scores. Finally, we re-trained our final model with the modified dataset under the same experimental conditions as the 1st model.

### Validating final model

We tested the validity of the final model by (1) comparing 20,040 predicted scores among diagnostic groups and (2) using an independent dataset of 50 participants. ANOVAs were conducted to confirm the differences among the diagnostic groups. For external validation, a total of 150 images (RCFT images from copy, immediate recall, and delayed recall) were scored separately by five experienced psychologists and our model. We designated the average scores of five human experts as the gold standard and compared the performance of six experts, including AI, with the gold standard.

## Results

### Characteristics of study participants

Table 1 shows the demographic characteristics and brief clinical information. In each clinical group, the relative proportions of females were 56.4%, 58.9%, and 47.6% for CN, MCI, and dementia, respectively. Dementia subjects had the highest mean ages (75.3), followed by MCI subjects (72.7) and CN subjects (71.9). Regarding education level, there was no significant difference between the CN and MCI groups (*p* > 0.05), but the dementia group had significantly lower educational levels (*p* < 0.01). Global cognition, measured by Mini-Mental Status Examination (MMSE), and RCFT scores differed significantly, and the lowest means were observed for the dementia group.

**Table 1:** Demographic and clinical characteristics of study participants

	**Total**	**CN**	**MCI**	**Dementia**
No. of participants	6680	4057	2331	292
Sex	3767 (56.4%)	2388 (58.9%)	1240 (53.2%)	139 (47.6%)
Age	72.3 (6.6)	71.9 (6.5)	72.7 (6.7)	75.3 (7.0)
Education	10.3 (4.6)	10.3 (4.5)	10.4 (4.6)	8.9 (5.0)
MMSE	26.4 (3.1)	27.2 (2.3)	25.7 (3.0)	20.3 (5.1)
RCFT [min, max]				
Copy	32.6 (4.5) [0, 36]	33.5 (3.2) [2.5, 36]	31.3 (5.4) [2.5, 36]	24.6 (9.6) [1, 36]
Immediate	13.7 (7.1) [0, 35.5]	15.5 (6.8) [0, 35.5]	10.4 (6.1) [0, 30]	4.5 (3.6) [0, 21]
Delayed	13.7 (6.7) [0, 34]	15.5 (6.3) [0, 34]	10.5 (5.8) [0, 32]	3.6 (3.7) [0, 17.5]

Data are presented as mean (standard deviation (SD)) for continuous variables and *N* (%) for the sex variable

*CN* Cognitive normal, *MCI* Mild cognitive impairment, *MMSE* Mini-Mental Status Examination

### Model performance

#### First model performance

**Fig. 3:**
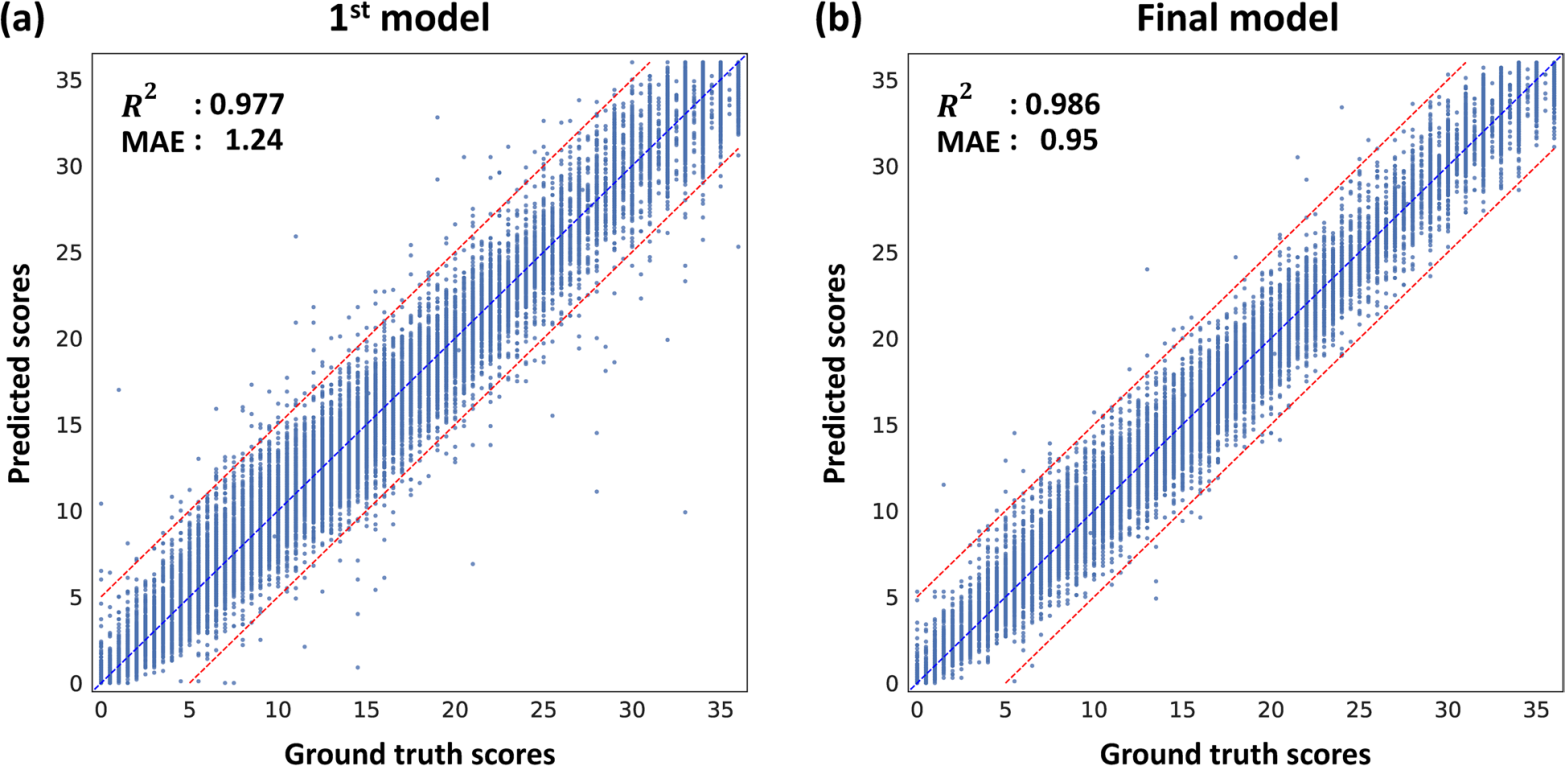
Scatter plots for 5-fold cross-validation. The results were considered by concatenating all results of 5-fold. For each scatter plot, the *x*-axis is the scores rated by psychologists, and the *y*-axis is the predicted scores from our model. The blue dot line is the line $y=x$, and the red dot lines are the lines $y=x \pm 5$. **a** A scatter plot for the 1st model. ${R}^{2}$: 0.977; mean absolute error (MAE): 1.24 (points). **b** A scatter plot for the final model. ${R}^{2}$: 0.986; MAE: 0.95

Figure 3a shows the accuracy of the RCFT scores predicted using the proposed model. Fivefold CV was conducted, and the predicted RCFT scores were compared with the ground truths. The MAE of the predicted scores was 1.24 points, and the correlation between the ground truths and predicted scores was ${R}^{2}=0.977$ ($r=0.988$). The MAEs of the predicted scores for the copy, immediate recall, and delayed recall images were 1.18, 1.21, and 1.30, respectively.

#### Model evaluation

We considered 20,040 images as the test set and compared their ground truths and predicted scores to validate the 1st model. We found that the differences between the ground truths and predicted RCFT scores were larger than five points for 188 images (0.9% of the total images). Among them, only 13 images had absolute differences of 10 or more points (0.06% of the total images) and absolute differences of the other 175 images (0.9% of the total images) belonged to 5 or more and 10 or less.

We grouped 188 images into four categories: one for quality error, 61 for scoring errors, 18 for digitalization errors, and 108 for model bias (Table 2). There were 175 images with absolute differences of more than five points and less than 10 points and consisted of one image quality error, 60 scoring errors, six digitalization errors, and 108 model biases. Thirteen images with absolute differences of 10 points or more were found and categorized into 12 digitalization errors and one scoring error.

**Table 2:** The error categorization for images with poor results

	5 ≤ absolute difference	10 ≤ absolute difference	5 ≤ absolute difference < 10
Total	188 (100%)	13 (100%)	175 (100%)
Image quality	1 (0.5%)	0 (0%)	1 (0.6%)
Scoring	61 (32.4%)	1 (7.7%)	60 (34.3%)
Digitalization	18 (9.6%)	12 (92.3%)	6 (3.4%)
Model bias	108 (57.5%)	0 (0%)	108 (62.7%)

#### Final model performance

We removed one image in the image error and re-entered the corrected scores for 79 images in the scoring and digitalization errors. But the images categorized into model bias were not modified. Figure 3b shows the prediction results of the final model. The model obtained MAE $=0.95$ (points) and ${R}^{2}=0.986$ ($r=0.993$), which suggested that the performance of the model improved after updating the model. Also, the results for each test were also consistent and slightly improved compared with the 1st model (MAE = 0.88, 1.12, and 0.85 for copy, immediate recall, and delayed recall, respectively).

### Validation of final model

#### Diagnostic validity

Predicted RCFT scores were compared among the CN, MCI, and dementia groups to test diagnostic validity. Predicted copy, immediate recall, and delayed recall scores were significantly different between all the pairs of the CN, MCI, and dementia groups (*p* < 0.01) (Table 3).

**Table 3:** Predicted RCFT scores in clinical groups

	**CN**	**MCI**	**Dementia**
No. of participants	4057	2331	292
Copy	33.3 (3.0)	31.3 (5.3)	24.8 (9.6)
Immediate	15.5 (6.7)	10.4 (6.1)	4.6 (3.6)
Delayed	15.4 (6.2)	10.6 (5.7)	3.7 (3.7)

Predicted 20,040 RCFT scores are presented as mean (standard deviation (SD))

*CN* Cognitive normal, *MCI* Mild cognitive impairment

#### External validity using independent sample

**Fig. 4:**
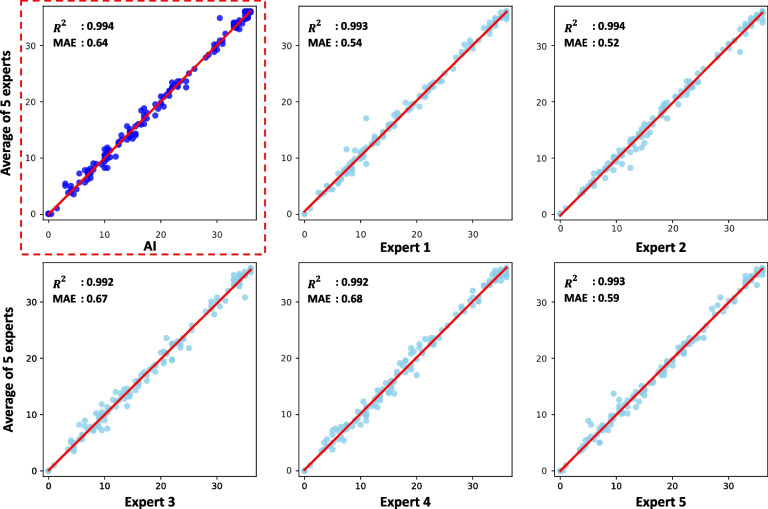
Scatter plots for comparisons of AI and 5 human experts. There were 150 RCFT drawings not used for prediction model building, and testing was scored by five human experts and AI. Scores of six experts including AI were compared with the gold standard (the average scores of five human experts). For each scatter plot, the *x*-axis represents the scores of each expert, the *y*-axis is the gold standard, and the red line is the line $y=x$

We compared the scores of six experts, including AI, with the gold standard (Fig. 4). The correlations between experts and the gold standard were almost the same (${R}^{2}$ for AI = 0.994; expert 1 = 0.993, expert 2 = 0.994, expert 3 = 0.992, expert 4 = 0.992, and expert 5 = 0.993). The MAEs of AI and five human experts with the gold standard were 0.64, 0.54, 0.52, 0.67, 0.68, and 0.59, respectively. Furthermore, the average of ${R}^{2}$ between scores by two different human experts was 0.983, but the average of ${R}^{2}$ between scores by AI and each expert was 0.988.

**Fig. 5:**
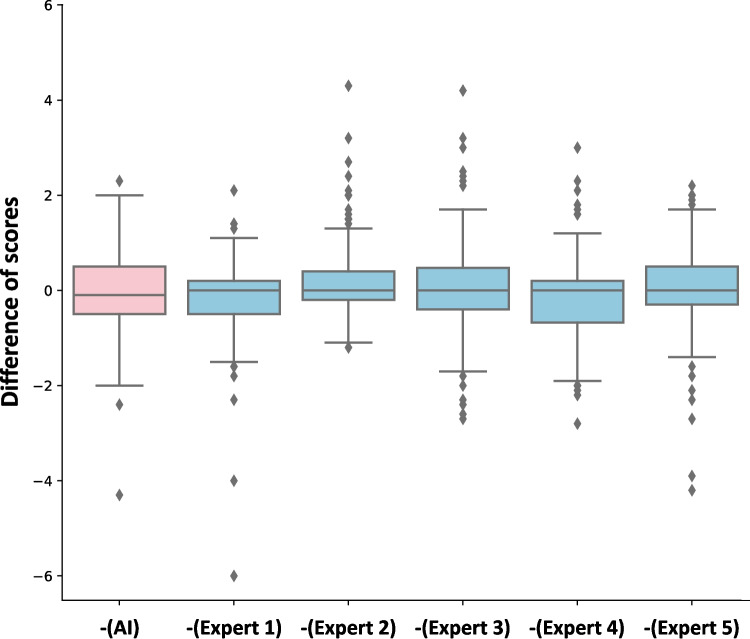
The boxplots of differences between scores by each expert including AI and the gold standard. Each boxplot is the boxplot of the score differences between each expert and the gold standard (the average scores of five human experts). For example, the boxplot for -(AI) (red boxplot) is the distribution of the score differences between AI and the gold standard, and -(E1) is the distribution of the score differences between expert 1 and the gold standard. AI: predicted scores of our model; E1: scores of expert 1; E2: scores of expert 2; E3: scores of expert 3; E4: scores of expert 4; E5: scores of expert 5

Figure 5 shows the differences between the scores of each expert, including the AI and the gold standard. For example, for expert 1, we calculated the score differences between the scores of expert 1 and the gold standard. The mean score differences were − 0.06, − 0.21, 0.21, 0.11, − 0.15, and 0.05 for AI and five human experts, and their standard deviations were 0.87, 0.86, 0.82, 1.00, 0.94, and 0.91, respectively. The accuracy of the scores predicted by AI was comparable to that of human experts.

## Discussion

The RCFT has been one of the useful neuropsychological tests in clinical and research settings. Here, we developed an automatic scoring system of 36 points using the DL model for the RCFT and confirmed its validity.

The proposed model had several distinctive features compared with previous studies. First, we firstly developed the automatic RCFT scoring system based on 36 points that was equivalent to the performance of human experts using the DL method. It takes only 10 s to score RCFT performance from the preprocessing to scoring. Rapid automation is one of the most significant benefits of this system in real-world clinical settings as well as research settings. Several studies have mainly focused on identifying the different patterns between cognitive impairment and normal cognition using digitalized RCFT images. However, little attention has been given to direct automatic scoring that was comparable to human experts’ scoring. Although several attempts have been made to develop the RCFT scoring systems, none of the studies has been reported scoring systems comparable to human experts and sufficiently validated [27, 28]. Generally, DL methods have been proven to outperform other methods in terms of prediction and improve generalization if a sufficiently large dataset was guaranteed [20, 31]. We utilized more than 20,000 images to train our DL model, which enabled the model to have a deeper architecture and capture the complex relationships between drawings and scorings of the RCFT.

Second, we tried to obtain data with good quality and further improve the quality of data as possible. All of our RCFT images were scored by 32 experienced psychologists. The psychologists in our study had specialized in neuropsychological assessment for dementia. Therefore, their scoring could be believed to be sufficient to serve as a gold standard for RCFT scoring. However, despite their delicate efforts, it was inevitable to encounter some noisy data (i.e., typos when digitalizing scores on the computer) for such a large dataset, which decreased the robustness of DL models [32, 33]. So, we improved the quality of the training data by re-examining some images so that we could increase the performance of our model. We developed the idea of active learning [34]. The initial model (1st model) built with the original data, including noisy data, was evaluated, and images with poor results (MAE ≥ 5 points) were re-examined. We updated our model by using the revised dataset and substantially improved the accuracy of the proposed method. Notably, these processes make our model more robust and reliable.

Third, applying AI systems in the medical field requires rigorous evaluation [35, 36]. Therefore, the validity of predicted RCFT scores using our model was verified in two ways. Diagnostic validity was confirmed that predicted scores differed between all pairs of the CN, MCI, and dementia groups. The result suggests that our automatic RCFT scoring might serve as one of the screening tools for cognitive impairment in the old population. Furthermore, we conducted an external validation with 150 images scored by five human experts. The average scores from five experts, not a single expert, were deemed to be the gold standard to increase the reliability of the evaluation. Our results revealed that the accuracy of AI was better than or like that of other experts based on the gold standard, even though the scores of each expert were included when calculating the gold standard. Furthermore, we confirmed that the average of ${R}^{2}$ between scores by AI and each human expert was better than the average of ${R}^{2}$ between scores by two different human experts, which indicated that our AI might be more accurate than experts on average. This external validation proved that our proposed model could be applied to the general population.

The validated automatic scoring system for the RCFT in the current study might increase the feasibility of the RCFT in a wide range of research and clinical fields. Also, it provided the first step toward the digital version of the traditional paper-and-pencil RCFT. Adapting traditional RCFT to tablet-based platforms might promote identifying individuals with very early stages of AD undetected in their communities. In particular, the RCFT delayed recall score showed significant predictability for tau pathology on the AD continuum [5]. Consequently, the RCFT using our automatic scoring system had the potential to allow community-based screening studies for AD pathology in a faster and more cost-effective way.

It is also worth noting that our automatic scoring system on the RCFT could be applied not only to AD or the elderly population but also to various clinical patient populations. Because we developed the automatic scoring system based on a wide range of scores (min–max score 0–36), it could cover diverse RCFT performances from neurological patients with different etiologies and ages. Moreover, the nonverbal nature of the test might make our automatic scoring system globally available.

Our study had some limitations and future directions for discussion. First, our model outputs only scores that did not represent the evidence for the predicted scores owing to the end-to-end nature of DL models. We plan to develop our model by separately predicting 18 scoring sections and summing the predicted scores of the 18 scoring sections. This approach makes it more human-like and increases the explainability of our model. Second, in the process of evaluating and updating the model, we only validated the images with absolute differences larger than five points between the ground truths and predicted scores. However, under limited resource, we tried to validate our training data and finally improved the performance. Third, we need to further evaluate the digital RCFT images using a digital pen. We are developing an application which examines RCFT with a tablet and outputs scores with our model. The tablet will enable a completely automated system from testing to scoring. However, to apply our model to digital images drawn by a digital pen that has quite different pen strokes from scanned images with a pencil, the additional test set with digital images will be further validated in our model. Moreover, digital pens can capture rich behavioral information such as organization patterns, pressure, velocity, and time in air and surface. As a result, this leads to the gathering hundreds of datapoints, and some of them could provide clinical significance. In the future, our product could be utilized for screening purposes for AD in medical checkup centers or in large-scale community-based research institutes.

## Conclusions

In conclusion, we developed an AI automatic scoring system for the RCFT based on the DL model with 20,040 images. We validated our model not only with a 5-fold CV but also with an independent test set. Our results suggested no fundamental difference between the rating scores of experienced psychologists and those of our automatic scoring system. We expected that our AI psychologist would be able to contribute to screen the early stage of AD pathology in medical checkup centers or large-scale community-based research institutes in a faster and cost-effective way.

## Data Availability

The dataset for the current study is not publicly available but is available from the corresponding author upon reasonable request.

## Abbreviations

AD: Alzheimer’s disease
CDT: Clock Drawing Test
CN: Cognitive normal
CNN: Convolutional neural networks
DL: Deep learning
FC: Fully connected
GAP: Global average pooling
MAE: Mean absolute error
MCI: Mild cognitive impairment
MMSE: Mini-Mental Status Examination
PDT: Pentagon drawing test
RELU: Rectified linear unit
RCFT: Rey Complex Figure Test
$${R}^{2}$$: *R*-Squared
